# Induction of polyploidy by nuclear fusion mechanism upon decreased expression of the nuclear envelope protein LAP2β in the human osteosarcoma cell line U2OS

**DOI:** 10.1186/1755-8166-7-9

**Published:** 2014-01-28

**Authors:** Shirley Oren Ben-Shoshan, Amos J Simon, Jasmine Jacob-Hirsch, Sigal Shaklai, Nurit Paz-Yaacov, Ninette Amariglio, Gideon Rechavi, Luba Trakhtenbrot

**Affiliations:** 1 Sheba Cancer Research Center, Chaim Sheba Medical Center, 52621, Tel-Hashomer, Israel; 2 Sackler Faculty of Medicine, Tel-Aviv University, Tel-Aviv, Israel; 3 Institute of Hematology, Chaim Sheba Medical Center, 52621, Tel-Hashomer, Israel

**Keywords:** LAP2, Cell fusion, Nuclear fusion, Polyploidy, Nuclear envelope, Osteosarcoma, SKY, U2OS

## Abstract

**Background:**

Polyploidy has been recognized for many years as an important hallmark of cancer cells. Polyploid cells can arise through cell fusion, endoreplication and abortive cell cycle*.* The inner nuclear membrane protein LAP2β plays key roles in nuclear envelope breakdown and reassembly during mitosis, initiation of replication and transcriptional repression. Here we studied the function of LAP2β in the maintenance of cell ploidy state, a role which has not yet been assigned to this protein.

**Results:**

By knocking down the expression of LAP2β, using both viral and non-viral RNAi approaches in osteosarcoma derived U2OS cells, we detected enlarged nuclear size, nearly doubling of DNA content and chromosomal duplications, as analyzed by fluorescent in situ hybridization and spectral karyotyping methodologies. Spectral karyotyping analyses revealed that near-hexaploid karyotypes of LAP2β knocked down cells consisted of not only seven duplicated chromosomal markers, as could be anticipated by genome duplication mechanism, but also of four single chromosomal markers. Furthermore, spectral karyotyping analysis revealed that both of two near-triploid U2OS sub-clones contained the seven markers that were duplicated in LAP2β knocked down cells, whereas the four single chromosomal markers were detected only in one of them. Gene expression profiling of LAP2β knocked down cells revealed that up to a third of the genes exhibiting significant changes in their expression are involved in cancer progression.

**Conclusions:**

Our results suggest that nuclear fusion mechanism underlies the polyploidization induction upon LAP2β reduced expression. Our study implies on a novel role of LAP2β in the maintenance of cell ploidy status. LAP2β depleted U2OS cells can serve as a model to investigate polyploidy and aneuploidy formation by nuclear fusion mechanism and its involvement in cancerogenesis.

## Background

The nuclear envelope (NE) is an essential structure of all eukaryotic cells that forms an interactive interface between the nucleus and cytoplasm, providing anchoring sites for chromatin domains at the nuclear periphery and enabling fundamental functions, such as DNA replication and RNA transcription to be carried out (reviewed in [1,2]). The NE is a double-unit membrane composed of an outer nuclear membrane (ONM), an inner nuclear membrane (INM), nuclear pore complexes (NPCs) and nuclear lamina. The lamins and their INM associating proteins, such as LBR, LAP2β, MAN1, emerin, and Nesprin-1α, were shown to be involved in most nuclear activities, including chromatin organization, DNA replication, transcription regulation, nuclear position, morphology and mechanical strength of the nucleus during interphase and governing NE breakdown and reassembly during mitosis [3-6]. It is now well accepted that these activities which are highly relevant to tumorigenesis, depending on the composition and organization of the NE [7].

LAP2β, the most ubiquitous and best studied LAP2 isoform, has been shown to execute some of these functions *via* its specific binding at the NE to lamin B1, BAF, GCL, HA95 and HDAC3 [8-11]. The binding of LAP2β to these partners enables exhibiting its key roles in nuclear envelope breakdown and reassembly during mitosis, initiation of replication and transcriptional repression. The latter activity was shown by us to occur as a result of LAP2β binding to chromatin modifiers, such as HDAC3, catalyzing gene silencing epigenetic modifications on histones ([11] and reviewed in [12]). The transcriptional repressive effect of LAP2β was evident on various cancer related transcription factors, including E2F5-DP3α, p53 and NFkB [10,11]. Another specific role of LAP2β in the regulation of nuclear lamina growth after the completion of NE reassembly and nuclear volume increase during the cell cycle was suggested earlier by Yang et al (1997) [13] in HeLa cells and by Gant et al (1999) [14] in *Xenopus laevis* extracts. In mitosis, for proper chromosomal segregation and in order to ensure that segregated DNA is enveloped in a single cell nucleus in each daughter cell, NE breakdown at early prophase and reassembly at late telophase are crucial phosphorylation dependent processes governed by the nuclear lamina, including LAP2β [15]. Indeed, it was shown by Anderson et al [16] that in U2OS cells reduced levels of LBR, LAP2β and MAN1 delayed and limited but not completely blocked NE formation in a manner consistent with built-in redundancy. Furthermore, over expression of these proteins accelerated NE formation, which caused a decrease in chromosome separation during mitosis [16]. These activities strongly link the nuclear lamina, including LAP2 proteins, to chromosomal stability in healthy cells. Recently it was shown that NE structural defects due to silencing of lamin A/C proteins caused chromosomal numerical instability and aneuploidy in ovarian cancer [17].

These observations led us to hypothesize that the absence of nuclear lamina components, such as LAP2β, may cause chromosomal instability and aneuploidy. In this study we investigated this aspect in U2OS cells taking the RNAi approach. We found that a reduction in LAP2β expression induced polyploidy by nuclear fusion mechanism, suggesting a novel role of an INM protein in polyploidy formation that may lead to cancer progression.

## Results

### LAP2β knocked down (KD) U2OS cells are characterized by doubled DNA content and centromeres duplication

**Figure 1:**
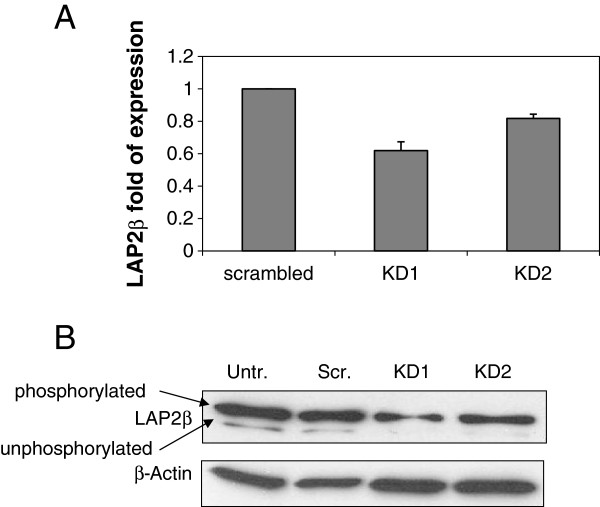
**LAP2β stable KD in individual U2OS clones.** LAP2β protein and RNA expression levels were evaluated by real time quantitative (RQ) PCR, normalized to importin (relative quantification) **(A)** and western blot analysis of nuclear proteins of U2OS untreated, scrambled and LAP2β KD clones 1 and 2 with mouse anti LAP2β and goat anti-β-Actin as loading control **(B)**.

**Figure 2:**
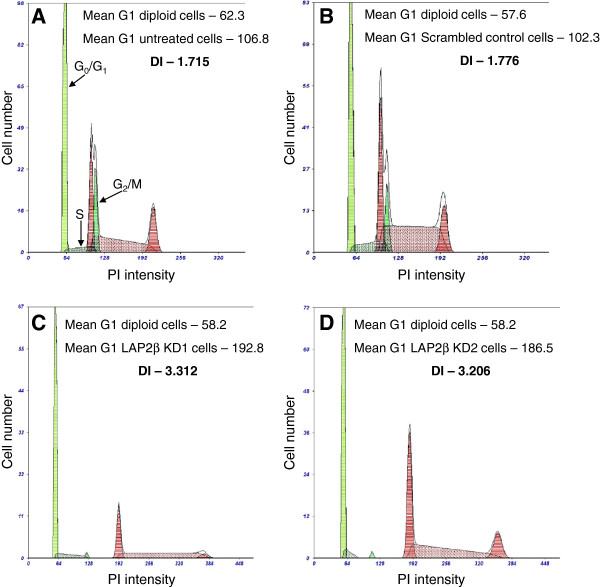
**DNA Index values determination by flow cytometry.** The histograms represent the percentage of cells in each phase of the cell cycle of U2OS cells (red) versus control normal diploid cells (green) and the Propidium Iodide (PI) fluorescence level. The final DI is calculated as the ratio between mean G_1_ fluorescence level in U2OS and normal cells. Doubled DI was measured in LAP2β KD clones 1 **(C)** and 2 **(D)** versus untreated **(A)**, scrambled control **(B)** U2OS cells.

The expression of LAP2β was stably knocked down in U2OS cells by both, retro-viral infection using the pSuper-retro vector (Figure 1) and non viral shRNA using pSuper vector (data not shown). In order to specifically reduce the expression of LAP2β and not the expression of the other LAP2 isoforms (α, γ, δ, ϵ and ζ), a specific shRNA oligonucleotide sequences were designed based on LAP2β specific sequence of LAP2 gene exon 6. Western blot analysis of LAP2β KD U2OS cells using the 6E10 mAb which is common to all LAP2 proteins revealed that indeed only LAP2β expression was reduced, without affecting the expression of the other isoforms (data not shown). LAP2β shRNA retro viral infected cells were grown for two weeks in a selective media before individual clones were isolated for further analyses. Two clones demonstrated significantly reduced LAP2β RNA (Figure 1A) and protein (Figure 1B) expression levels. Both clones were investigated for DNA content and karyotype analyses. DNA index (DI) analysis was performed by flow cytometry to measure the DNA content of the cells. DIs of 3.312 and 3.206 were found in LAP2β KD clones 1 and 2, respectively, almost double than 1.715 and 1.776 that were obtained in untreated and scrambled cells, respectively (Figure 2). This indicates that LAP2β KD cells underwent duplication of DNA content as a result of LAP2β reduced protein level. In order to eliminate the possibility that the nearly doubled DI of the LAP2β KD clones is due to binucleated U2OS cells, we examined the cell morphology of these clones, compared with untreated and scrambled cells. As can be seen in Additional file 1: Figure S1, binucleated cells were not present in control cells as well as in LAP2β KD clones.

**Figure 3:**
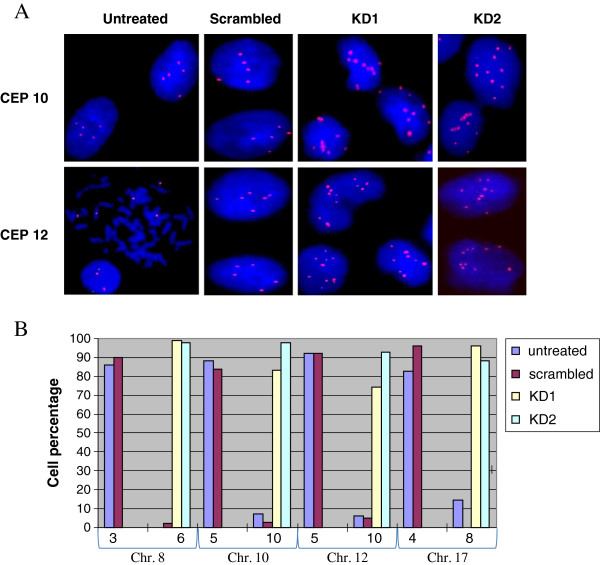
**Centromere signal numbers upon LAP2β KD in U2OS cells. (A)** FISH hybridization pattern of centromeres of chromosomes 10 and 12. **(B)** Quantification of centromeric signal numbers of the same chromosomes, based on analyses of 300 cells in each sample.

We next performed fluorescence *in situ* hybridization (FISH) to examine the observed DNA content duplication, using centromeric probes for chromosomes 8, 10, 12 and 17. Duplication of modal centromeres numbers was demonstrated for all tested chromosomes (Figure 3). Small population (≤10%) of cells with duplicated centromere numbers also were found in untreated and scrambled control U2OS cells, a phenomenon which is documented in the literature for this cell line [18]. It is important to note, that we observed, using FISH, the same DNA duplication using the non- viral RNAi approach, reinforcing our hypothesis that polyploidy was obtained as a consequence of LAP2β reduced level (Additional file 2: Figure S2). The polyploid phenotype was not observed by us when the expression of LAP2β was reduced using the RNAi approach in another cancer cell line, HepG2, as well as in the diploid primary arising retinal pigment epithelia (ARPE) cells (Additional file 3: Figure S3).

### Karyotypes of LAP2β depleted cells consist of duplicated and single chromosomal markers

**Figure 4:**
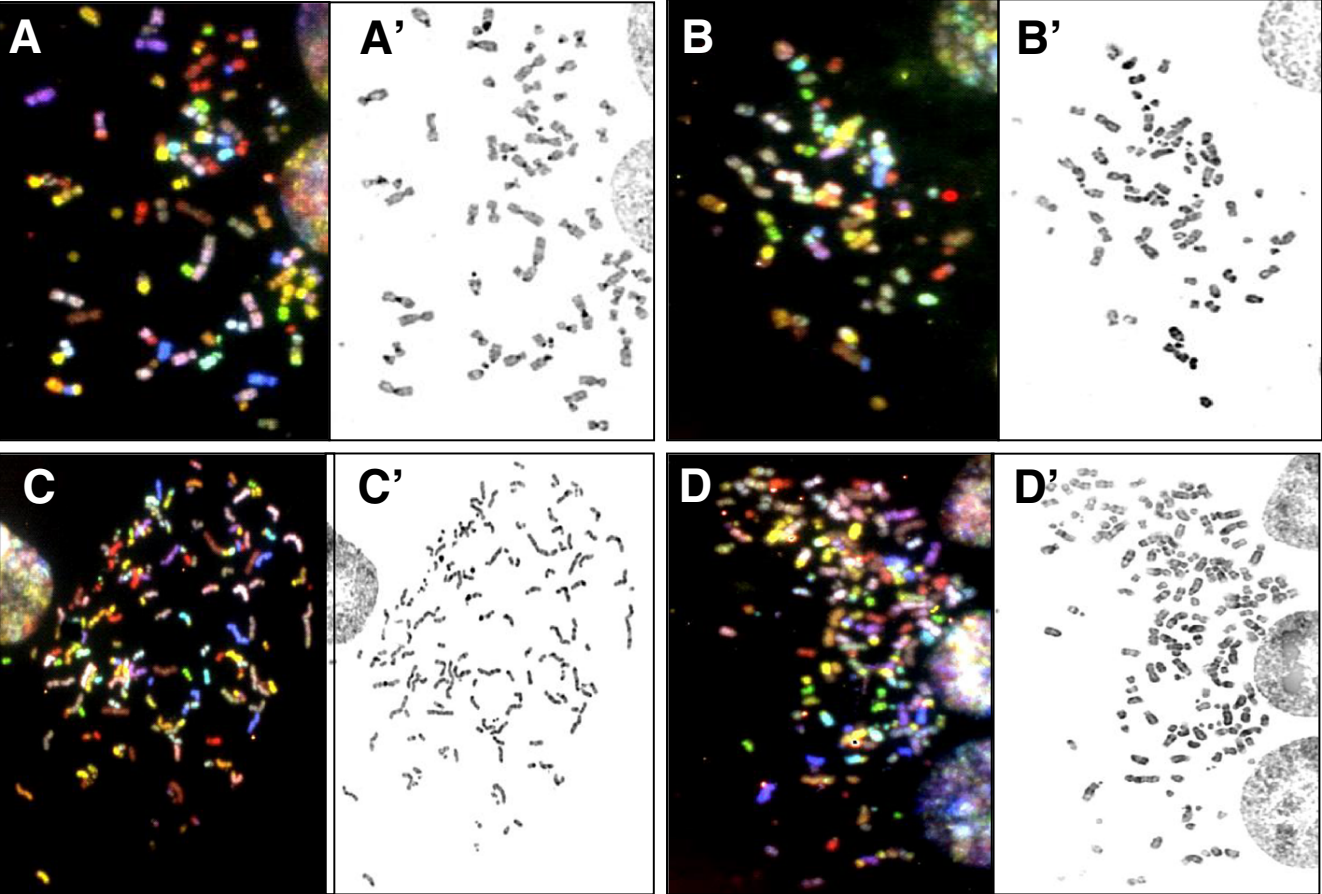
**Duplication of chromosomes number in U2OS LAP2β KD cells.** Representative SKY images (RGB display) from untreated **(A)**, scrambled control **(B)**, LAP2β KD clone 1 **(C)** and 2 **(D)**. A’-D’–the inverted DAPI images.

**Figure 5:**
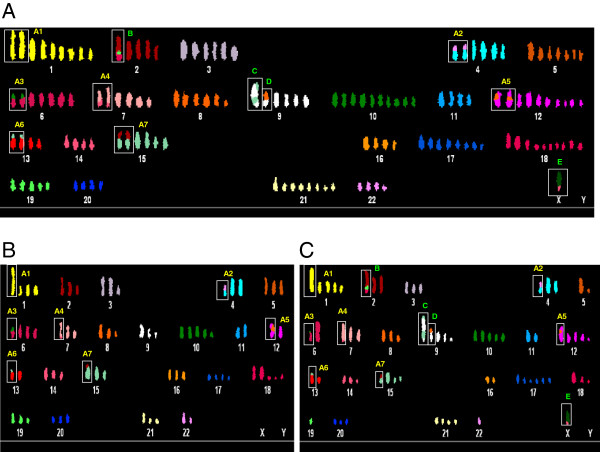
**Duplicated and single chromosomal markers in LAP2β KD karyotypes.** Representative karyotypes from LAP2β KD clone 1 **(A)** and two sub-clones of untreated **(B, C)** U2OS cells (U2OS-1 and U2OS-2, respectively). The frames are around chromosomal markers: A1-A7 (yellow) which are common in all the sub-clones and are duplicated upon LAP2β KD; B-E (green) which are typical only for one sub-clone and stay as single in LAP2β KD cells. Chromosome numbers (white) are indicated.

To evaluate whether the chromosomal duplication phenotype of LAP2β KD cells represent a phenotype of the entire genome in these cells, we chose to use spectral karyotyping (SKY) analysis. Comparison between chromosome numbers per metaphase revealed about two fold duplication upon LAP2β KD. Near-triploid metaphases of untreated and scrambled control consisted an average of 70 ± 5.48 (range 58-79) and 69 ± 4.13 (range 57-78) chromosomes, respectively, whereas LAP2β KD near-hexaploid metaphases consisted an average of 138 ± 7.32 (range 127-150) and 136 ± 6.41 (range 131-150) chromosomes, in LAP2β KD1 and KD2 clones, respectively (Figure 4). The wide range of chromosome number in each clone resulted also from the presence of small chromosomes which contained centromeres, a phenomenon that was reported in other cytogenetic studies [18]. The classification of these centromeres according to DAPI banding and SKY image is complicated, therefore in our karyotype characterizations we did not assign them as clear chromosomal markers. The SKY analyses showed that the karyotypes of untreated and scrambled U2OS cells was characterized by high level of structural and numerical chromosomal alterations, a phenomenon which is described in the literature for this cell line [19]. We therefore focused on clear, well defined chromosomal markers, where the involvement of specific chromosomes was confirmed by FISH using whole chromosome painting probes. The SKY analyses showed that the karyotypes of LAP2β KD clones 1 and 2 are characterized by seven double chromosomal markers (Figure 5A, A1-A7 in yellow), namely, der(1)t(1;21)×2, der(4)t(4;22)×2, der(6)t(6;10)×2, der(7)t(7;14)×2, der(8)t(8;12)×2, der(13)t(13;15)×2 and der(15)t(15;2)×2, and four single chromosomal markers (Figure 5A, B-E in green), namely der(2)t(2;19;18), der(9)t(9;15), der(9)t(9;8) and der(X)t(X;18). Furthermore, SKY analysis revealed that the both characterized U2OS sub-clones, U2OS-1 and U2OS-2, contained the seven markers that were duplicated in LAP2β KD, but the four single chromosomal markers were detected only in U2OS-2 (Figure 5C) and did not exist in the U2OS-1 (Figure 5B).

### Microarray analysis identifies differentially expressed genes in LAP2β KD cells

**Figure 6:**
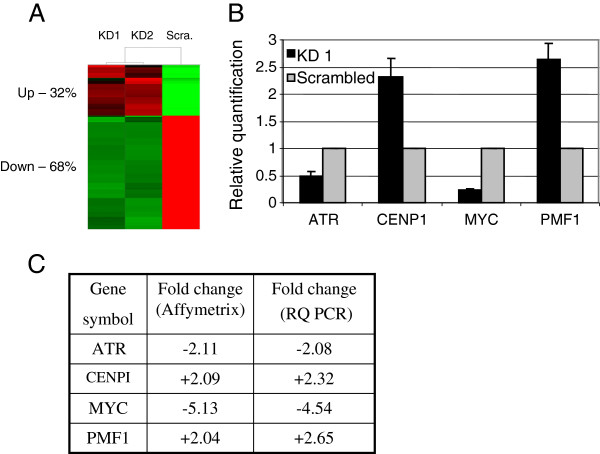
**Differentially expressed genes in LAP2β KD cells. A**. The cluster analysis represents the 342 genes that were significantly down (green) or up (red) regulated by two fold or more in both LAP2β KD U2OS clones. **B**. Validation of microarray results by RQ-PCR. Four genes were assayed in triplicates for each sample. All values were normalized to the reference gene RPLPO. **C**. Comparison table of the expression values of the four validated genes as obtained by microarray and RQ-PCR analyses.

To elucidate changes in gene expression in U2OS cells upon LAP2β KD, microarray expression profiling of Human transcriptome (Affymetrix GENE1.0 ST oligonucleotide array) was performed. Comparison of gene expression profiles between LAP2β KD clones and scrambled control yielded a list of 342 genes that exhibited significantly 2 fold down or up-regulation in both LAP2β KD U2OS clones (Figure 6A, Additional file 4: Table S1). The expression profile of 4 of the changed genes, ATR, MYC, CENP1 and PMF1 was validated by qPCR (Figure 6B) and was found to be consistent with the levels detected by the microarray analysis (Figure 6C). The 342 gene list was analyzed for functional annotation clustering using two bioinformatic analysis tools, Ingenuity and DAVID. The top functional ‘Ingenuity’ categories that were most significantly affected in LAP2β KD cells are summarized in Table 1. They included ‘cell cycle’ and ‘DNA replication, recombination, and repair’ groups which are relevant to chromosomal integrity and maintenance of ploidy (Additional file 4: Table S2). The most significant category of disease, consisting of the highest number of genes, was found to be cancer (Table 2, Pv = 1.58E-06, 108 genes). 31.6% of the genes in this group are involved in aspects of worsening and progression of cancer, such as apoptosis (Pv = 4.40E-04), colony formation (Pv = 2.02E-06), invasion (Pv = 2.23E-05) and tumorigenesis (Pv = 9.17E-06).

**Table 1:** Top functional categories, significantly changed (p < 0.05), ‘Ingenuity’ analysis

	**Category**	**Functions**	**No. genes**	**P-value**
1	Organismal functions	Healing	11	5.42E-08–4.70E-03
2	Cellular movement	cell movement, chemotaxis, homing, intravasation, invasion, migration	54	1.58E-06–8.04E-03
3	Cellular growth and proliferation	colony formation, growth, hypertrophy, proliferation	81	2.02E-06–8.28E-03
4	Cell to cell signaling and interaction	attachment, recruitment, activation, adhesion, penetration	30	1.09E-05–8.36E-03
5	Hair and skin development and function	cell movement, development, migration	7	2.05E-05–2.87E-03
6	Cell death	apoptosis, cell death, survival	69	2.08E-05–8.16E-03
7	Skeletal and muscular system development and function	adhesion, cell movement, development, differentiation, formation, migration, proliferation	20	2.49E-05–6.34E-03
8	Cellular development	developmental process/disorder, differentiation, growth, hypertrophy, tubulogenesis	52	4.66E-05–8.37E-03
9	Cell cycle	cell division process, cell stage, G0/G1 phase transition, G1/S phase transition, G2/M phase transition, interphase, mitosis	25	5.60E-05–7.53E-03
10	Tumor morphology	growth, invasion, proliferation, regression, size	17	9.06E-05–7.33E-03
11	DNA replication, recombination and repair	synthesis, metabolism of DNA	24	1.23E-04–6.94E-03
12	Organismal survival	death, survival	42	1.27E-04–3.27E-03
13	Immune response	accumulation, cell movement, proliferation	29	1.73E-04–8.04E-03
14	Immune and lymphatic system development and function	accumulation, development, differentiation, growth, mitosis, morphology, proliferation, quantity, size	33	1.73E-04–7.91E-03
15	Tissue development	accumulation, adhesion, developmental process, formation	20	1.73E-04–8.15E-03
16	Embryonic development	differentiation, quantity, tubulogenesis	14	1.86E-04–4.70E-03
17	Organismal development	angiogenesis, developmental process, neovascularization, size	40	2.62E-04–2.92E-03
18	Gene expression	activation, binding, expression, stabilization, transactivation, transcription	39	2.65E-04–8.08E-03

**Table 2:** Genes distribution according to their involvement in diseases (p < 0.05)

	**Diseases**	**No. genes**	**P-value**
1	Cancer	108	1.58E-06–8.16E-03
2	Reproductive system disease	58	9.12E-06–5.65E-03
3	Skeletal and muscular disorders	41	1.03E-05–8.02E-03
4	Gastrointestinal disease	42	1.47E-05–8.01E-03
5	Hematological disease	32	1.77E-05–7.52E-03

## Discussion

LAP2β, the major LAP2 family nuclear membrane isoform, is mainly recognized for its essential roles in transcriptional repression at the NE (reviewed in [12]) and promotion of NE breakdown and reassembly during mitosis [20]. Here we show by using the RNAi approach that individual U2OS clones stably depleted of LAP2β are characterized by duplicated DNA content and number of chromosomes, as was documented by flow cytometry, FISH and cytogenetic analyses. These surprising findings demonstrate the involvement of an INM protein in the maintenance of cell ploidy status.

**Figure 7:**
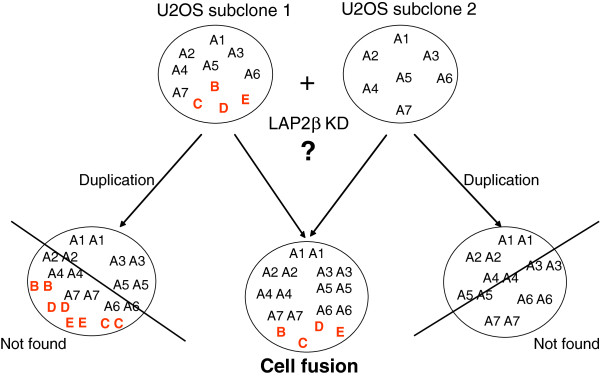
**Schematic representation of the possible mechanisms producing the polyploid cells upon LAP2β KD.** A1-A7 are common chromosomal markers that exist in two copies upon LAP2β KD. B-E are typical chromosomal markers that stay as single copy in LAP2β KD cells.

An increased number of whole chromosome sets, polyploidy, especially in its simplest tetraploidy(4n) form, is highly prevalent in different forms of cancer, particularly in the early stages, suggesting a role for this phenomenon in cancer promotion [21,22]. It has been proposed that tetraploid state is an unstable intermediate, which can result in aneuploidy and cancer (reviewed in [23]). It was shown that mouse tetraploid cells generated by the process of telomere-driven tetraploidization are more tumorigenic then their diploid counterparts [24]. Mouse model of ovarian cancer provides direct evidence for “diploidy-tetraploidy-aneuploidy” pathway: tetraploid cells arising from cytokinesis failure of diploid cells display higher rates of chromosome mis-segregation, compared to their diploid progenitors, leading to the generation of numerous aneuploid daughter cells [25]. Polyploid cells in a diploid organism can be formed by three general mechanisms: cell fusion, endoreplication and abortive cell cycle including cytokinesis failure and mitotic slippage [26]. We asked which of these mechanisms can explain the near-hexaploid karyotype of our LAP2β depleted U2OS cells. SKY analysis of these cells revealed that two sets of chromosomes from two distinct nuclei were jointed. Karyotyping of untreated U2OS cells revealed two different near-triploid sub-clones that can be distinguished by specific chromosomal markers. Analysis of LAP2β KD karyotypes revealed near duplicated number of chromosomes that included two copies of common chromosomal markers (A1-A7, Figure 5) and only a single copy of chromosomal markers that are unique to U2OS-2 sub- clone (B-E, Figure 5). The fact that not all the chromosomal markers were duplicated leads us to reject the possibility that the polyploid phenotype resulted from whole genome duplication. The chance for whole genome duplication to occur followed by loss of a combination of chromosomal markers that characterize the sub clone, together with the fact that the same combinations of chromosomal markers appeared in different polyploid cells, is very low, thus ruling out this possibility (Figure 7).

The concept that cell fusion contributes to tumorigenesis was proposed more than 100 years ago. In recent years, a potentially important role of cell fusion in the tumor progression, including acquisition of metastasis ability, generation of cancer stem cells and multidrug resistance was suggested [27-31]. It was shown that when leukemia cells fuse with stromal cells *in vivo,* the malignant potential of the leukemia cells is maintained in the resulting hybrid cells that allow assuming of fusion as a mechanism of gene transfer for cancer dissemination [32]. Moreover, it was found that fusion and genomic hybridization between cancer and bone marrow derived cells play an important role in the generation of metastasis [33]. Based on our study we hypothesise that an aberrant expression of LAP2β in cancer cells, such as osteosarcoma, which U2OS cells derived from, may lead under certain circumstances to the fusion of these cancer cells, either with themselves to promote local tumor or with bone marrow derived cells to form metastases.

**Figure 8:**
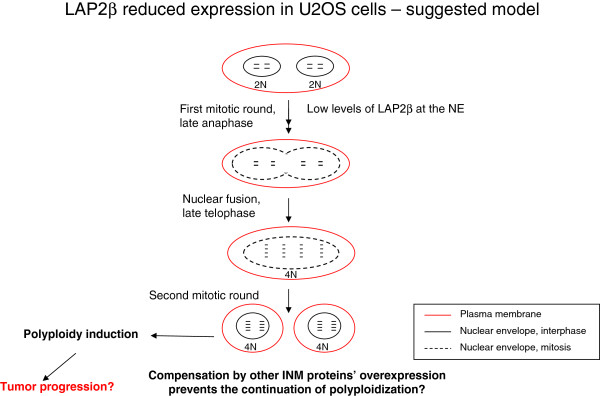
Suggested model for polyploidization by nuclear fusion mechanism in U2OS LAP2β KD cells.

We suggest that the process of fusion between two distinct nuclei is associated with and rely on the reassembly of the NE at the end of mitosis. LAP2β has a pivotal role during mitosis in governing NE breakdown and reassembly [20,34]. In late anaphase the two new NEs start to form around each segregated mass of chromatin, such that nuclear reassembly is completed in telophase (for review see [15]). Hetzer and his colleagues [16] showed that NE reformation timing is important for proper cell cycle progression and is coordinated with other mitotic events in anaphase/telophase. Moreover, decreasing the expression levels of LAP2β, LBR or MAN1 in U2OS cells delayed NE reformation, while the over expression of these proteins accelerated this process and interfered with chromosomal segregation during mitosis. Knocking down the expression of one of the INM proteins could be rescued by the over expression of a different chromatin binding INM counterpart or by increasing BAF or LBR protein levels. The fact that reduction of one of the INM proteins is unable to completely block NE formation is indicative for a redundant system in which NE reassembly is accomplished even in the absence of one of the proteins, possibly at a slower rate [16]. Considering this, and our obtained results, we propose a model (Figure 8) suggesting that in LAP2β KD fused U2OS cells there is a delay in NE reassembly which allows the fusion of nuclear contents of the fused cells before two new NEs are formed around the two nuclei of the fused cells. In this case, cytokinesis event is abolished because only one nucleus is formed and enveloped. However, due to INM redundancy in the process of NE reassembly, at the next mitotic round, the loss of LAP2β may be compensated by other INM LEM proteins, such as LBR, MAN1 and emerin, or by lamin B1 and other proteins of the nuclear lamina, allowing NE breakdown and reassembly to be carried out undisturbed.

According to our suggested model (Figure 8), despite a compensation mechanism at the NE, the LAP2β depleted cells remain in an irreversible polyploid phenotype because of the initial damage. It is also possible that nuclear fusion events continue to occur, resulting in a greater chromosomal instability and aneuploidy, not compatible with living cells in the culture, driving them to cell death. This may imply on a unique role for LAP2β in guarding the cell from unwanted fusion of two distinct nuclei contents, an initial event that can lead to tumorigenesis. Our hypothesized link between LAP2β depletion, polyploidization and the resulting cancer progression in osteosarcoma derived cells is reinforced by the microarray results. Further functional analysis of the specific genes that were expressional deregulated upon LAP2β knock down are needed to substantiate this link. Interestingly, the polyploid phenotype was not observed by us when the expression of LAP2β was reduced in another non-diploid cell line, HepG2, and in the diploid primary ARPE cells. We speculate that a specific genetic or epigenetic background existing in U2OS, and not in the other two examined cell lines, predispose these cancerous cells to enhanced nuclear fusion, leading to polyploidy upon additional cellular aberrations, such as reduced expression of LAP2β at the NE.

Finally, regardless of LAP2β, our results may imply that in tetraploid/polyploidy tumor cells, when single rearranged chromosomes are found [19,35-39] it may be the result of fusion between two heterogeneous cancer cells, rather than the formation of chromosome rearrangement, occurring on the tetraploid/polyploidy per se. Therefore, we hypothesize that in the progression of cancer, cell fusion events are much more frequent than suggested previously. We suggest not to rule out the possibility of cell fusion mechanism when chromosomal changes are cytogenetically analyzed during cancer progression.

## Conclusions

The presented results in this study suggest that nuclear fusion mechanism underlies the polyploidization induction upon LAP2β reduced expression in U2OS cells. Our study implies on a novel role of an INM protein, LAP2β, in the maintenance of cell ploidy status in U2OS cells. Thus, LAP2β depleted U2OS cells can serve as a model to investigate polyploidy and aneuploidy formation by nuclear fusion mechanism and its involvement in cancer initiation and progression. Such a cellular model can also enhance our understanding of nuclear fusion mechanism and the importance of nuclear envelope reassembly timing at the end of mitosis.

## Methods

### Cell lines

The human osteosarcoma cell line U2OS , embryonic kidney (HEK293T) and hepatocellular carcinoma HepG2 cells were cultured in Dulbecco’s modified Eagle’s medium (DMEM) (Biological Industries, Israel) supplemented with 10% fetal calf serum (FCS), 2 mM glutamine, 100 mg/ml streptomycin and 100 units/ml penicillin (Biological Industries). Human retinal pigmented epithelium (ARPE) cells were cultured with a 1:1 mixture of Dulbecco’s modified Eagle’s medium and Ham’s F12 medium (Sigma Aldrich) with 3 mM glutamine, 10% fetal bovine serum. All cells were grown at 37°C in a humidified incubator with 5% CO2.

### Transfection and viral infection

pSuper shRNA retro-viral (a generous gift from Prof. Shai Izraeli, Sheba cancer research center, Tel-Hashomer, Israel) and non-viral (a generous gift from Prof. Reuven Agami, The Netherlands Cancer Institute, Amsterdam, The Netherlands) systems were used to generate LAP2β stable knocked down (KD) in the U2OS cell line. shRNA oligonucleotides sequences were designed based on LAP2β specific sequence of exon 6, according to standard rules of shRNA synthesis:

Forward 5′-GATCCCCCTGAGACTGAATGGACAAGTTCAAGAGACTTGTCCATTCAGTCTCAGTTTTTGGAAA-3′, Reverse 5′- AGCTTTTCCAAAAACTGAGACTGAATGGACAAGTCTCTTGAACTTGTCCATTCAGTCTCAGGGG -3′.

Virions for infection were produced by co-transfection of 293T cells with the targeting LAP2β plasmid or non-targeting scrambled plasmid and packaging proteins (pMD2G plasmid for Gag-Pol proteins and pCGP for Env) using FUGENE6 transfection reagent (Roche Diagnostics) according to manufacturer’s instructions. 48 hours post the first infection, 2 μg/ml Puromycin (Sigma) was added for selection. The surviving cells were grown for two weeks in selection media and were analyzed for LAP2β KD using western blot and RQ-PCR analyses.

### Nuclear protein extraction

Untreated, scrambled control and LAP2β KD U2OS cells were plated at 10^6^ cells in a 10 cm plate. Cells were harvested, washed twice with cold PBS, pelleted and resuspended and incubated in 400 μl of an ice-cold hypotonic buffer (10 mM Hepes pH 7.9, 10 mM KCl, 1 mM EDTA, 1 mM EGTA, 0.1 M DTT and protease inhibitors (‘Complete’, Roche Molecular Biochemicals, Manheim, Germany)) for 15 min. on ice. 25 μl of 10% NP-40 were added and cells were vortexed vigorously for 10 sec. and pelleted at 14,000 RPM for 1 min. at 4°C. The supernatant containing the cytoplasmatic proteins was separated from the pelleted nuclear fraction, taken into a different tube. The pelleted nuclear fraction was resuspended in 200 μl of ice-cold lysis buffer (20 mM HEPES pH 7.9, 0.4 M NaCl, 1 mM EDTA, 1 mM EGTA, 0.1 M DTT and protease inhibitors) and was mixed vigorously for 15 min. at 4°C. Samples were centrifuged at 14,000 RPM for 5 min. at 4°C and the nuclear supernatant was removed to a different tube. Protein concentration of each sample was determined by Bradford modified method (BCA Protein Assay kit, Pierce, Rockford, IL, USA) and equal amounts of proteins were subjected to Western blot analysis.

### Western blotting

Proteins were separated on a 10% SDS-PAGE gel (Invitrogen, USA) in tris glycine running buffer, transferred to a nitrocellulose membranes using iBlot® Dry Blotting System (Invitrogen) and detected using the ECL chemiluminescence reagent plus (Pharmacia Biotech, NJ, USA). The following primary antibodies were used: mouse monoclonal anti LAP2β (clone 6G11, dilution 1:2500) and polyclonal goat anti β-actin (1:1000, Santa Cruz Biotechnology). Secondary antibodies used were peroxidase-conjugated goat anti-mouse and peroxidase-conjugated donkey anti-goat (Jackson Immuno-Research Laboratories, USA) diluted 1:10,000 and 1:5,000, respectively.

### RNA extraction and Reverse transcription PCR

Total RNA was extracted from LAP2β KD, scrambled and untreated U2OS cells that were grown in 10 cm culture dishes to a confluence of 90%, using TRIzol reagent (invitrogen) according to manufacturer’s instructions. First-strand cDNA was generated from 2 μg of total RNA in the presence of random hexamer primers using M-MLV reverse transcriptase (Invitrogen) according to manufacturer’s instruction.

### Real-time quantitative PCR

Gene expression was quantified by real-time PCR (RQ-PCR) using SYBR green PCR Master Mix (Applied Biosystems) for LAP2β and Importin (control reference gene). The primers used are as follows (5′ to 3′, final concentration 500 nM): Importin forward: TGGGCCCTCTCATATCTATCA, Importin reverse: CCACTTTATAATCATTATGCA, LAP2β forward: CGGACCTCTGCAGGCATTAAC, LAP2β reverse: TTATAGTTTCAGCTATGGGAGTACTCTCTG. All the reactions were performed in triplicates using the ABI Prism 7900 SDS instrument (Applied Biosystems). Relative gene expression values were determined using the 2^-ddCt^ method according to the manufacturer’s instruction.

### DNA index (DI) analysis by flow cytometry

DNA content of U2OS nuclei was measured on a FACScan flow cytometer (Becton-Dickinson, Sunnyvale, CA). DIs were determined by mixing the cells of interest with normal human diploid leukocytes, followed by propidium iodide (PI) staining (Beckman coulter, USA). PI as a fluorescent dye binds stoichiometrically to the DNA so the fluorescence data is considered as a measurement of the cellular DNA content. Normal diploid cells behave as reference to identify the position of cells with normal diploid amount of DNA [40]. According to PI staining of U2OS and normal diploid mix cells, cell cycle histograms of each cells population with the fluorescence emitted level in each phase (G_0_/G_1_, S, G_2_/M) were obtained. DI of each sample of U2OS cells is calculated as the fluorescence level of G_0_/G_1_ U2OS cells relative to the G_0_/G_1_ peak of normal diploid cells.

### Fluorescence in situ hybridization (FISH)

Directly labled centromeric DNA probes for chromosomes 8, 10, 12 and 17 (Vysis, Downers, Grove, IL and Kreatech, Amsterdam, The Netherlands) and whole chromosome painting probes (Applied Spectral imaging (ASI), Migdal HaEmek, Israel) for chromosomes 2,7,8,9,10,15,18 and 19 were hybridized according to the manufacturer’s instructions. At least 300 cells were analyzed in each sample.

Slides were analyzed using an Olympus IX81 fluorescence light microscope (Olympus, Tokyo, Japan) equipped with a Plan Apo objective 100x/1.4 oil, an appropriate spectral filter [BH2-TFC1 Triple Band filter DAPI (4,6-diamidino 2-phenyl-indole)/FITC (fluorescein isothiocyanate)/TRITC (tetramethylrhodamine isothiocyanate)], and a 100 W mercury arc lamp.

### Spectral karyotyping (SKY)

SKY was performed with the SKY fluorescent labeling kit (Applied Spectral Imaging, Migdal HaEmek, Israel) according to the manufacturer’s protocol. Chromosomes were counterstained with DAPI. Image acquisition was performed by use of an SD200 Spectracube (Applied Spectral Imaging) mounted on an Olympus BH-2 microscope using a custom-designed optical filter (SKY-1, Chroma Technology, Brattleboro, VT). Automatic identification of chromosomes was based on the measurement of the spectrum for each chromosome as described previously [36]. At least 20 metaphase cells were analyzed for each of U2OS untreated, scrambled and LAP2β KD clones.

### Microarray hybridization and data analysis

Experiments were performed using Affymetrix GeneChip Human Gene 1.0 ST Arrays according to manufacturer’s recommendations. Briefly, 100-600 ng of total RNA from LAP2β KD clones and scrambled control cells was used to generate first-strand cDNA using random hexamers primer. After second-strand synthesis, *in vitro* transcription was performed. The resulting cRNA was then used for a second cycle of first-strand cDNA with UTP resulting in single-stranded DNA which was used for fragmentation and terminal labeling. cDNA generated from each sample was processed as per manufacturer’s recommendation using an Affymetrix GeneChip Instrument System manual (https://www.affymetrix.com/support/downloads/manuals/wt_sensetarget_label_manual.pdf).

Gene level RMA sketch algorithm (Affymetrix Expression Console and Partek Genomics Suite 6.2) was used for crude data generation. Genes were filtered according to a two fold cutoff up or down regulation and analyzed using unsupervised hierarchical cluster analysis (Spotfire DecisionSite for Functional Genomics; Somerville, MA) to get a first assessment of the data. Further processing includes functional analysis and over-representation calculations based on Gene Ontology and DAVID [41,42]. Over-representation calculations were done using Ease (DAVID) and Ingenuity IPA software builds protein interaction networks (Ingenuity® Systems, http://www.ingenuity.com). Data results are deposited in http://www.ncbi.nlm.nih.gov/geo/.

## Abbreviations

NE: Nuclear envelope; ONM: Outer nuclear membrane; INM: Inner nuclear membrane; FISH: Fluorescent in situ hybridization; SKY: Spectral karyotyping; KD: Knock down; DI: DNA index.

## Competing interests

The authors declare that they have no competing interests.

## Authors’ contribution

SOB designed the study, carried out the viral knock down experiments, the cytogenetic experiments and the cellular and molecular studies, analyzed the data, drafted the manuscript and wrote its final version of the manuscript. AJS designed the study, participated in the knock down experiments, analyzed the data, drafted the manuscript and wrote its final version. JJH carried out the microarray experiment and analyzed its data. SS carried out the non-viral knock down experiments. NPY analyzed the gene expression data. NA designed the study, analyzed the data and drafted the manuscript. GR designed the study, analyzed the data and drafted the manuscript. LT designed the study, participated in the cytogenetic experiments, analyzed the data, drafted the manuscript and wrote its final version. All authors read and approved the final manuscript.

## Supplementary Material

Additional file 1: Figure S1 U2OS cells morphology. Light microscopy observation of untreated, scrambled control and LAP2β KD U2OS cells. All images were taken using X10 magnification. Click here for file

Additional file 2: Figure S2 FISH analysis of non-viral LAP2β shRNA transfected U2OS cells. FISH pattern using whole chromosome probes of chromosomes 2, 3, 17 and 18 in untreated (Unt.) and LAP2β KD cells. Click here for file

Additional file 3: Figure S3 FISH analysis of chromosome numbers upon LAP2β KD in HepG2 and ARPE cells. A - western blot analysis of HepG2 nuclear protein extracts of untreated, scrambled and two LAP2β KD clones using mouse anti LAP2β mAb (6G11 clone). β-Actin was used for equal loading control (I). FISH pattern of centromeres of chromosomes 10 and 12 (II). B–western blot analysis of ARPE nuclear protein extracts of untreated and two LAP2β KD clones using mouse anti LAP2β mAb (6G11 clone) (III). B–FISH pattern using whole chromosome 3 painting probe (IV). Click here for file

Additional file 4: Table S1 A complete list of significantly up and down regulated genes in LAP2β KD U2OS cells. Microarray analysis was used to compare relative transcript levels between LAP2β KD clones and scrambled U2OS cells as described in material and methods. The table lists 342 genes that were up or down regulated at least 2 fold in both KD clones. Data is provided as fold change in gene expression, relative to scrambled control. **Table S2.** List of functional groups and genes related to cell fusion in LAP2β KD U2OS cells. Bioinformatic analyses were performed using ‘Ingenuity’ and ‘DAVID’ algorithms. Click here for file
