# Supplementary figures and images for: Induction of polyploidy by nuclear fusion mechanism upon decreased expression of the nuclear envelope protein LAP2β in the human osteosarcoma cell line U2OS
Source: Mol Cytogenet. 2014 Jan 28;7:9. doi: 10.1186/1755-8166-7-9 (PMC3926685; doi:10.1186/1755-8166-7-9)

## Slide 1
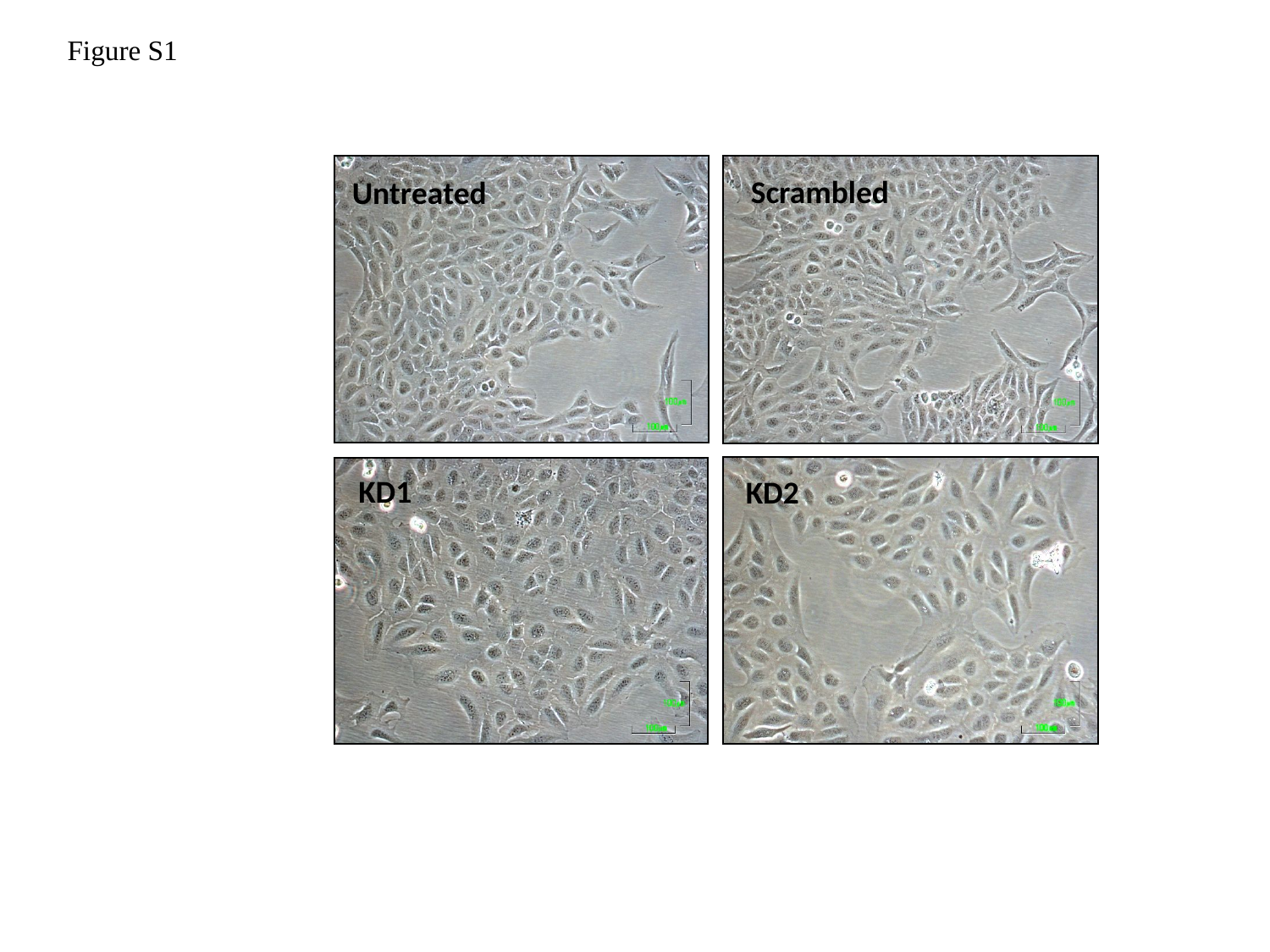

Figure S1
Scrambled
Untreated
KD1
KD2

Supplement: Additional file 1: Figure S1 — U2OS cells morphology. Light microscopy observation of untreated, scrambled control and LAP2β KD U2OS cells. All images were taken using X10 magnification. [file 1755-8166-7-9-S1.pptx]

## Slide 1
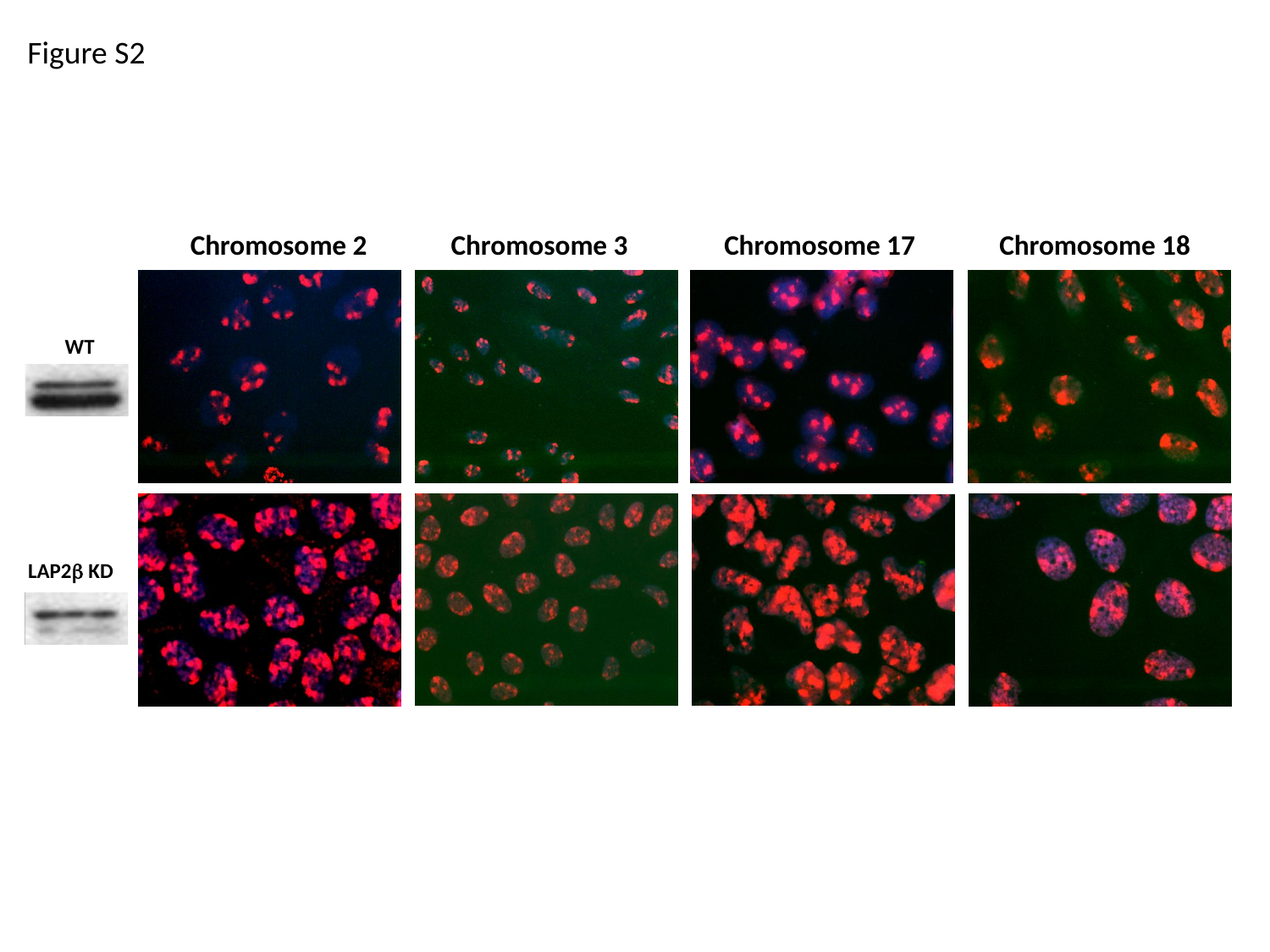

Figure S2
Chromosome 2
Chromosome 3
Chromosome 17
Chromosome 18
WT
LAP2b KD

Supplement: Additional file 2: Figure S2 — FISH analysis of non-viral LAP2β shRNA transfected U2OS cells. FISH pattern using whole chromosome probes of chromosomes 2, 3, 17 and 18 in untreated (Unt.) and LAP2β KD cells. [file 1755-8166-7-9-S2.pptx]
