# Supplementary material for: Induction of polyploidy by nuclear fusion mechanism upon decreased expression of the nuclear envelope protein LAP2β in the human osteosarcoma cell line U2OS
Source: Mol Cytogenet. 2014 Jan 28;7:9. doi: 10.1186/1755-8166-7-9 (PMC3926685; doi:10.1186/1755-8166-7-9)
Supplement: Additional file 3: Figure S3 — FISH analysis of chromosome numbers upon LAP2β KD in HepG2 and ARPE cells. A - western blot analysis of HepG2 nuclear protein extracts of untreated, scrambled and two LAP2β KD clones using mouse anti LAP2β mAb (6G11 clone). β-Actin was used for equal loading control (I). FISH pattern of centromeres of chromosomes 10 and 12 (II). B–western blot analysis of ARPE nuclear protein extracts of untreated and two LAP2β KD clones using mouse anti LAP2β mAb (6G11 clone) (III). B–FISH pattern using whole chromosome 3 painting probe (IV). [file 1755-8166-7-9-S3.ppt]

## Slide 1
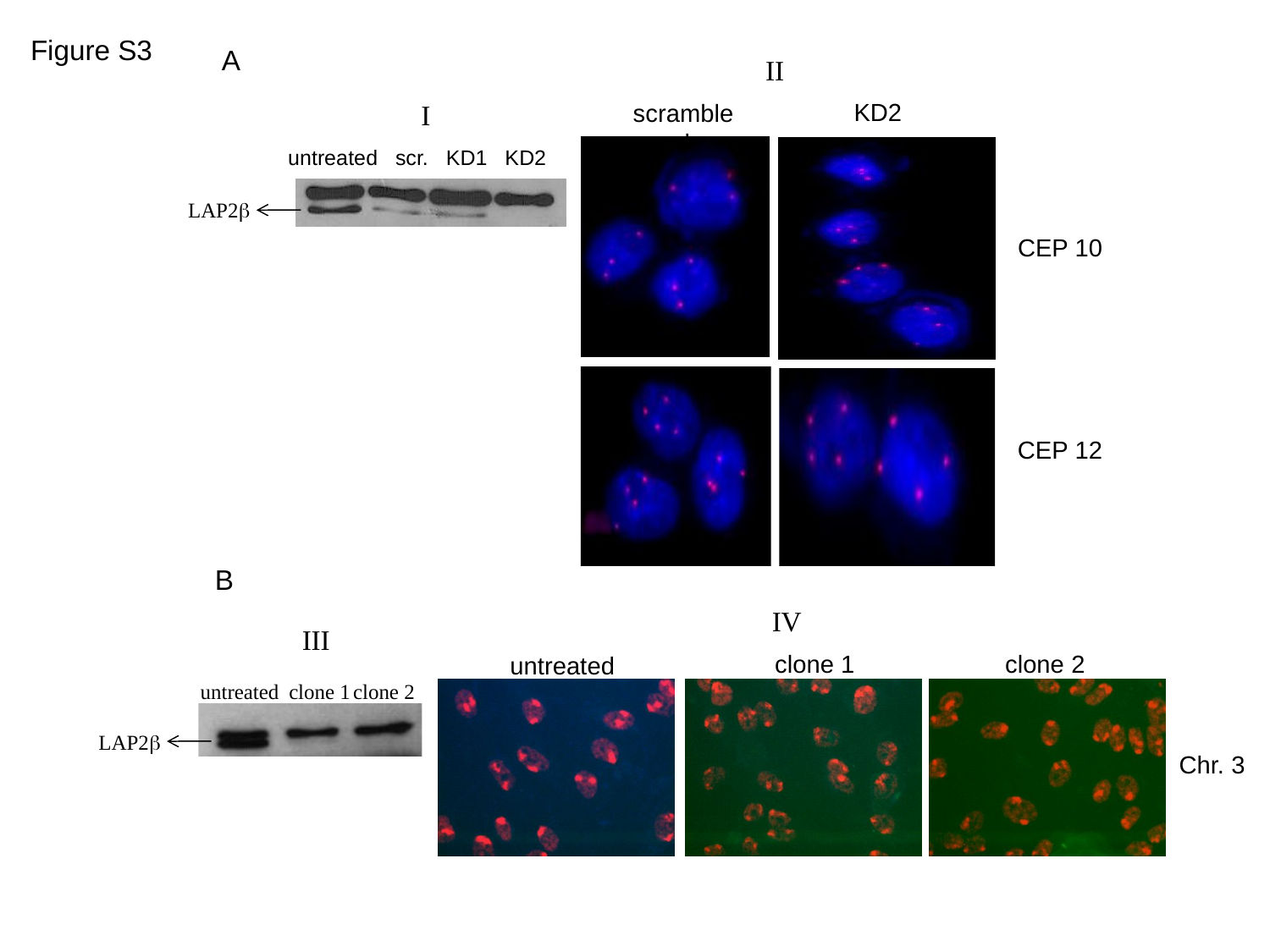

Figure S3
A
II
KD2
I
scrambled
untreated scr. KD1 KD2
LAP2β
CEP 10
CEP 12
B
IV
III
clone 1
clone 2
untreated
Chr. 3
untreated
clone 1
clone 2
LAP2β
